# Effect of benzo(a)pyrene on oxidative stress and inflammatory mediators in astrocytes and HIV-infected macrophages

**DOI:** 10.1371/journal.pone.0275874

**Published:** 2022-10-14

**Authors:** Asit Kumar, Namita Sinha, Sunitha Kodidela, Lina Zhou, Udai P. Singh, Santosh Kumar

**Affiliations:** Department of Pharmaceutical Sciences, College of Pharmacy, The University of Tennessee Health Science Center, Memphis, TN, United States of America; University of Miami, UNITED STATES

## Abstract

**Background:**

Benzo(a)pyrene (BaP), an important polycyclic aromatic hydrocarbons (PAH) component of cigarette/tobacco smoking, is known to cause adverse health effects and is responsible for various life-threatening conditions including cancer. However, it is not yet clear whether BaP contributes to the macrophage- and astrocyte-mediated inflammatory response.

**Methods:**

We examined the acute (up to 72 h) effects of BaP on the expression of antioxidant enzymes (AOEs), cytokines/chemokines, and cytochromes P450 (CYP) enzymes in astrocytic cell lines, SVGA, and chronically HIV-infected U1 macrophage. The treated cells were examined for mRNA, protein levels of CYPs, AOEs superoxide dismutase-1 (SOD1) and catalase (CAT), cytokines/chemokines, using Western blot, multiplex ELISA, and reactive oxygen species (ROS) by flow cytometry analysis.

**Results:**

Upon acute exposure, BaP (1 μM) showed a significant increase in the mRNA levels of CYPs (CYP1A1 and CYP1B1), and pro-inflammatory cytokine IL-1β in SVGA cells following BaP for 24, 48, and 72h. In addition, we observed a significant increase in the mRNA levels of SOD1 and CAT at 24h of BaP treatment. In contrast, BaP did not exert any change in the protein expression of AOEs and CYP enzymes. In U1 cells, however, we noticed an interesting increase in the levels of MCP-1 as well as a modest increase in TNFα, IL-8 and IL-1β levels observed at 72 h of BaP treatment but could not reach to statistically significant level.

**Conclusions:**

Overall, these results suggest that BaP contributes in part to macrophage and astrocyte-mediated neuroinflammation by mainly inducing IL-1β and MCP-1 production, which is likely to occur with the involvement of CYP and/or oxidative stress pathways.

## Introduction

Cigarette smoking is known to cause the accumulation of toxic metabolites and induction of oxidative stress and can contribute to the onset or progression of several diseases including chronic obstructive pulmonary disease (COPD) [1–4], and coronary heart disease (CHD) [5–7], and cancer [8–10]. Smoking can initiate a series of events that include induction of inflammatory cytokines/chemokines, altering antioxidant enzymes (AOEs), and metabolism of smoking constituents through dysregulation of cytochromes P450 (CYP) enzymes [11–13]. Nicotine is the most studied constituent of tobacco and one of the extensively used substances among substance or drug users [14]. In our previous studies, we have shown that nicotine induces oxidative stress through the CYP2A6-mediated pathway in U937 monocytic cells and SVGA cells, an immortalized astrocytic cell line [11, 15]. Moreover, BaP, a known carcinogenic polycyclic aromatic hydrocarbon (PAHs) present in cigarette smoke, has been shown to increase the expression of CYP1A and AOEs superoxide dismutase-1, (SOD1) and catalase (CAT), resulting in reactive oxygen species (ROS) generation and cytotoxicity in U937 cells [16].

Glial cells including astrocytes are important to brain cells that mediate effects in the brain upon tobacco smoking, perhaps via CYP and oxidative stress pathways, not only in HIV-infected but also in general populations [17, 18]. Astrocytes are not primarily infected with HIV but are possible through cell-cell contact and receptor-mediated endocytosis, however, they may become activated as a result of proximity to HIV-infected macrophages and microglia [19, 20]. SVGA cells have been extensively studied to show the effect of astrocyte activation and subsequent neuroinflammation [15, 21, 22]

Similar to inflammation, the expression of AOEs and CYPs might also be altered by smoking and tobacco-related products. Thus, there is a need to identify the constituent(s) of tobacco and the underlying mechanism that is responsible for smoking-associated inflammation and oxidative stress in astrocytes. We hypothesize that BaP, a major carcinogen in a cigarette can influence the expression of specific cytokines/chemokines and AOEs (SOD1 and CAT) in astrocytes, that may be associated with smoking-associated toxicity and progression of inflammatory conditions in smokers. Further, we have shown that BaP enhances HIV replication in differentiated U1 macrophages by a CYP-mediated oxidative stress pathway [23]. But to date, it is not yet clear whether BaP contributes to the macrophage and brain resident astrocyte-mediated inflammatory response. Therefore, the objective of this study was to determine the effect of BaP on the acute inflammatory and oxidative stress mediators in the astrocytic and HIV-infected differentiated U1 macrophages.

## Materials and methods

### Cell culture and treatment

The human SVGA astrocytic cell line was maintained as previously discussed [15]. In brief, SVGA cells were cultured in DMEM supplemented with 10% (v/v) Fetal bovine serum (FBS), 1% penicillin, and streptomycin (40 U/ml and 40 mg/ml, respectively) at 37°C with 5% CO_2_. Astrocytes were treated with BaP (0.01–1 μM, Millipore Sigma, Cat No. B1760) every 24 h for up to 72 h for acute treatment. A stock solution of BaP was prepared in 100% dimethyl sulfoxide (DMSO; Millipore Sigma. The final concentration of DMSO in the cell culture media was not more than 0.3%. BaP concentration was maintained constant at every treatment.

Chronically infected HIV‐promonocytic (U1) cell lines were obtained from the NIH AIDS Reagent Program (Germantown, MD). In this study, we used U1, instead of uninfected U937 cells, because U937 cells are non-HIV and have already been characterized for oxidative stress and inflammatory regulation in the presence of tobacco constituents [11, 16]. It’s now important to study HIV-infected macrophages (U1), which are juxtaposed to astrocytic cells and could activate astrocytic cells during HIV infection in the brain. The cells were cultured in RPMI 1640 media containing 10% (v/v) FBS. 1% penicillin, and streptomycin (40 U/ml and 40 mg/ml, respectively) at 37°C with 5% CO_2_ [23]. To differentiate the cells into macrophages, 0.8 million cells were seeded in 1.5 ml of media containing 100 nM phorbol 12-myristate 13-acetate (PMA) in each well of a 6-well plate. After 3 days, the media containing PMA and non-adherent cells were removed and the differentiated cells were washed with phosphate buffer saline. The cells were topped with fresh 1 ml media and treated with different concentrations of BaP (0.01–1 μM) every 24 h for up to 72 h for acute treatment. BaP concentration was maintained constant at every treatment.

### Western blotting

For western blot, cells were lysed with RIPA buffer and cellular protein was quantified using Pierce BCA Protein Assay Kit (ThermoFisher Scientific). Each sample was loaded onto 10% SDS-PAGE gel and the rest of the procedures were performed as described previously [13, 24]. We evaluated the expression of proteins associated with AOEs (SOD1 and CAT), pro-inflammatory cytokine (IL-1β), and CYP enzymes (CYP1A1, CYP1B1, CYP2A6) and quantified by Western blot. Following SDS-PAGE, proteins were transferred onto PVDF membrane and blocked in Li-Cor blocking buffer (LI-COR Biosciences, Lincoln, NE) for 1 h. After blocking, membranes were incubated overnight at 4°C with primary antibodies, including SOD1 mouse mAb (1:500 dilution, catalog no. sc-101523, Santa Cruz Biotechnology), and CAT mouse mAb (1:500 dilution, catalog no. sc-365738, Santa Cruz Biotechnology), IL-1β rabbit pAb (1:1000 dilution, catalog no. 16806-1AP, Proteintech), CYP2A6 rabbit mAb (1:1000 dilution, catalog no. ab3570, Abcam), CYP1A1 mouse mAb (1:500 dilution, catalog no. sc-48432, Santa Cruz Biotechnology), CYP1B1 mouse mAb (1:500 dilution, catalog no. sc-374228, Santa Cruz Biotechnology), and β-Actin mouse mAb (1:1000 dilution, catalog no. 3700, Cell Signaling). The next day, after washing (three times for 5 min each in PBS containing 0.2% Tween 20), membranes were incubated with either goat anti-mouse or goat anti-rabbit secondary antibody (1:10000 dilution, LI-COR Biosciences) for 1 h at room temperature in the dark. After 1 h membranes were washed and scanned using the Li-Cor Scanner (LI-COR Biosciences). Densitometry analyses of the proteins were performed using LI-COR Image Studio Software (v.5.2, Nebraska, USA).

### LDH activity

The cytotoxic effect of BaP in U1 and SVGA cells was evaluated by LDH release using the CyQUANT™ LDH Cytotoxicity Assay Kit (Catalog no. C20300, ThermoFisher Scientific) according to the manufacturer’s instruction. Briefly, U1 and SVGA cells were exposed to BaP (0.01–1 μM) for 24, 48, and 72 h. Cell culture supernatants were collected at indicated time points and transferred to a 96-well plate. The reaction mixture was added to the cell culture supernatant for 30 min at room temperature. Background absorbance 680 nm was subtracted from 490 nm absorbance to determine the LDH activity.

### Measurement of reactive oxygen species (ROS)

ROS was quantified by flow cytometry analysis using the fluorescence dye 5-(and-6)-chloromethyl 2’,7’- dichlorodihydrofluorescein diacetate (CM-H_2_DCFDA) (ThermoFisher Scientific). The treated cells were thoroughly washed with PBS and resuspended in 5μM of CM-H_2_DCFDA in PBS. The cells were then incubated at room temperature in the dark for 45 minutes in CM-H_2_DCFDA and subsequently washed and resuspended in 300 μL of PBS. ROS generated in the cells was detected and data was analyzed using flow cytometry built-in software (Agilent NovoCyte).

### mRNA Real-Time PCR

RNA was isolated using an RNeasy mini kit (Qiagen). For cDNA synthesis, total RNA was reverse transcribed using the High-Capacity cDNA Reverse Transcription Kit (ThermoFisher Scientific) as previously described [25]. In brief, every 20 ml of reverse transcription (RT) reaction consisted of 2 ml of 10 RT buffer, 0.8 ml of 25 dNTP mix (100 mM), 2.0 ml of 10 RT random primers, 1.0 ml of MultiScribe reverse transcriptase, 4.2 ml of nuclease-free water, and 10 ml of the RNA sample. Thermal cycling conditions used for reverse transcription are 10 minutes at 25°C, 120 minutes at 37°C, and finally 5 minutes at 85°C followed by a stop at 4°C. The synthesized cDNA was the template for the real-time PCR amplification that was carried out by Quant Studio 6 Pro real-time PCR System (ThermoFisher Scientific), using TaqMan Universal Master Mix II, no UNG (ThermoFisher Scientific) as per the company’s protocol. Reaction conditions were 10 minutes at 95°C, followed by 40 cycles of 15 seconds at 95°C, 60 seconds at 60°C, followed by the final plate read. The following primers were used in this study: Hs00533490_m1 SOD1 (human), Hs00156308_m1 CAT (human), Hs00868409_s1 CYP2A6 (human), Hs01054794_m1 CYP1A1 (human), Hs00164383_m1 CYP1B1 (human), Hs01555410_m1 IL-1β (human), and Hs99999903_m1 Actin (human). Actin served as an internal control for sample normalization and the comparative cycle threshold method (2^-ΔΔCt^) was used for data quantification as described previously [26, 27].

### Multiplex ELISA

The levels of cytokines (pro-inflammatory: TNF-α, IL-1β, IL-6, IL-8, IL-18; anti-inflammatory: IL-1RA, IL-10) and chemokines (MCP-1 and RANTES) were measured in the cell culture supernatant using customized human 9-Plex ProcartaPlex^TM^ multiplex immunoassay (ThermoFisher Scientific) as per previously described method [13]. Briefly, SVGA cells were treated with BaP (1 μM) for 24, 48, and 72 h. Cell culture supernatants were collected at indicated time points and transferred to a 96-well ELISA plate. The samples and standards were incubated with a magnetic 96-well ELISA plate for 1 h at room temperature. Afterward, wells were washed, and subsequently, detection antibody, streptavidin-PE, and reading buffer were added followed by a plate read. Quantification was achieved as per the manufacturer’s kit operating manual. The concentration of cytokines and chemokines was expressed as pg/ml.

### Statistical analysis

The data were analyzed using Graph-Pad version 5.0 (GraphPad Software Inc., San Diego, CA, USA). Values of all experiments are represented as mean ± SEM of at least three independent experiments. Values were compared using Student’s t-test (two groups) or one-way ANOVA with Tukey’s test (multiple comparisons), when appropriate. The level of significance was set at *p ≤0.05, **p ≤0.01, ***p ≤0.001.

## Results

### Effect of BaP on AOEs and IL-1β expression in U1 macrophages

**Fig 1:**
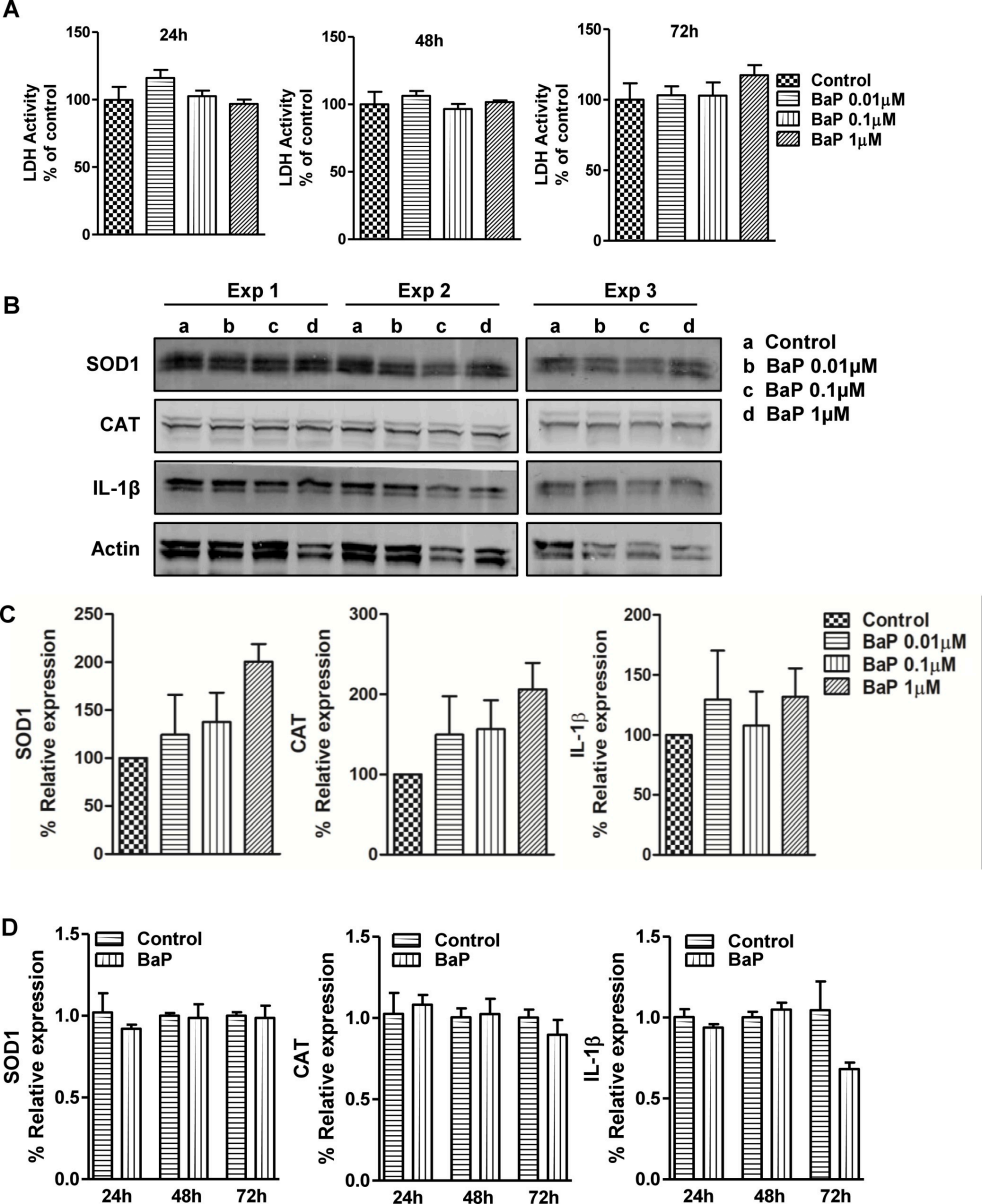
LDH activity and protein expression of AOEs and cytokine IL-1β (A, B). (A) LDH activity was performed in U1 cells treated with different concentrations of BaP (daily treatment up to 72 h). (B) Representative Western blots of AOEs (SOD1 and CAT) and IL-1β in U1 cells (Western blot at 72 h) treated with different concentrations of BaP (daily treatment up to day 3). Untreated controls were considered as 100% in the densitometry analysis. (C) Densitometry analysis. (D) mRNA expression was analyzed in U1 cells (at 24, 48, and 72 h) treated with 1 μM concentration of BaP (daily treatment). Statistical analyses were carried out by using one-way ANOVA (multiple comparison) and Student’s t-test (two groups). Results are expressed as means ± S.E.M of n = 3–4 experiments.

Differentiated U1 cells were treated with different concentrations of BaP (0.01, 0.1, 1 μM) every 24 h up to 72 h to determine the effect of BaP on AOEs (SOD1 and CAT) and pro-inflammatory cytokine (IL-1β) (Fig 1). Before analyzing AOEs and IL-1β, we performed an LDH assay to analyze the cytotoxicity of BaP on U1 cells. We observed that treatment of U1 cells with BaP for 24, 48, and 72 h did not show a statistically significant increase in LDH activity (Fig 1A), indicating no detectable cytotoxicity with the selected doses of BaP. Next, we performed a Western blot and observed an upward trend in the protein expression of SOD1, CAT, and IL-1β with increasing concentrations of BaP at 72 h (Fig 1B and 1C, S1 Fig). However, none of these could reach a statistically significant level. To evaluate whether BaP exerts effects at the transcriptional level on SOD1, CAT, and IL-1β, U1 cells were treated with 1 μM BaP for 24, 48, and 72 h (Fig 1D). Similar to protein expression, BaP did not significantly affect the SOD1, CAT, and IL-1β gene expression in U1 cells even at different time points.

### Effect of BaP on Reactive Oxygen Species (ROS) in U1 macrophages

**Fig 2:**
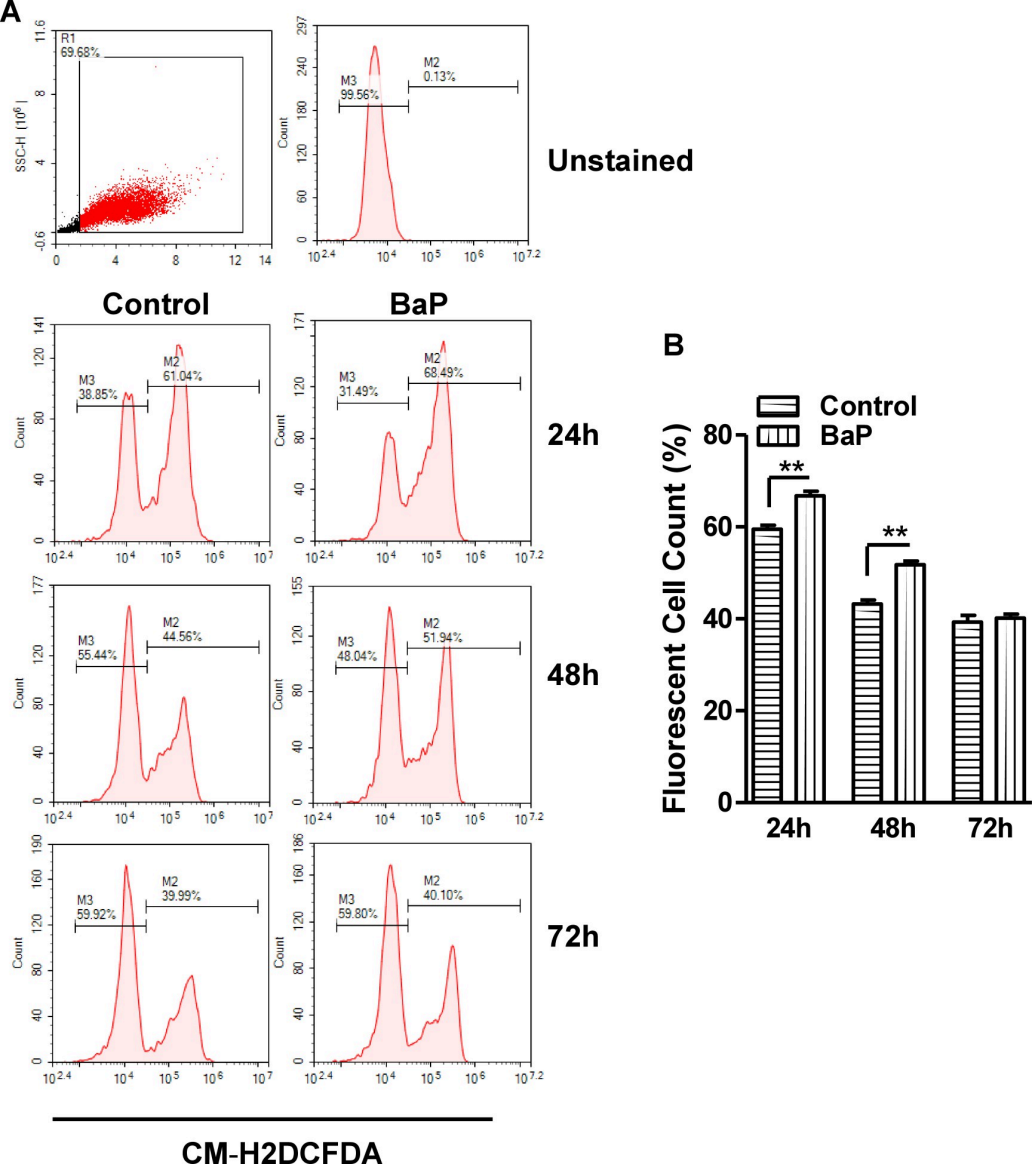
Effect of BaP on ROS in U1 cells (A, B). (A) The U1 cells were treated with 1 μM BaP for 24, 48, and 72 h. ROS level using flow cytometry. (B) Data is quantified using florescent cell count that were measured in %. Statistical analyses were carried out by using Student’s t-test (two groups each time point). Results are expressed as means ± S.E.M of n = 3 experiments. **P ≤0.01 in comparison with untreated control.

To study the effect of BaP on ROS, U1 cells were treated with 1 μM BaP for 24, 48, and 72 h. We noticed that 1 μM does show maximum effect without any toxicity is the reason to use this dose. In the flow cytometry analysis, we observed a significant increase in the ROS with BaP treatment at 24 and 48 h (Fig 2A and 2B). However, there was not much effect on ROS with BaP concentration at 72 h time point. This might be due to the threshold level of ROS with time or other factors that required further investigations.

### Effect of BaP on cytokines/chemokines in U1 macrophages

**Fig 3:**
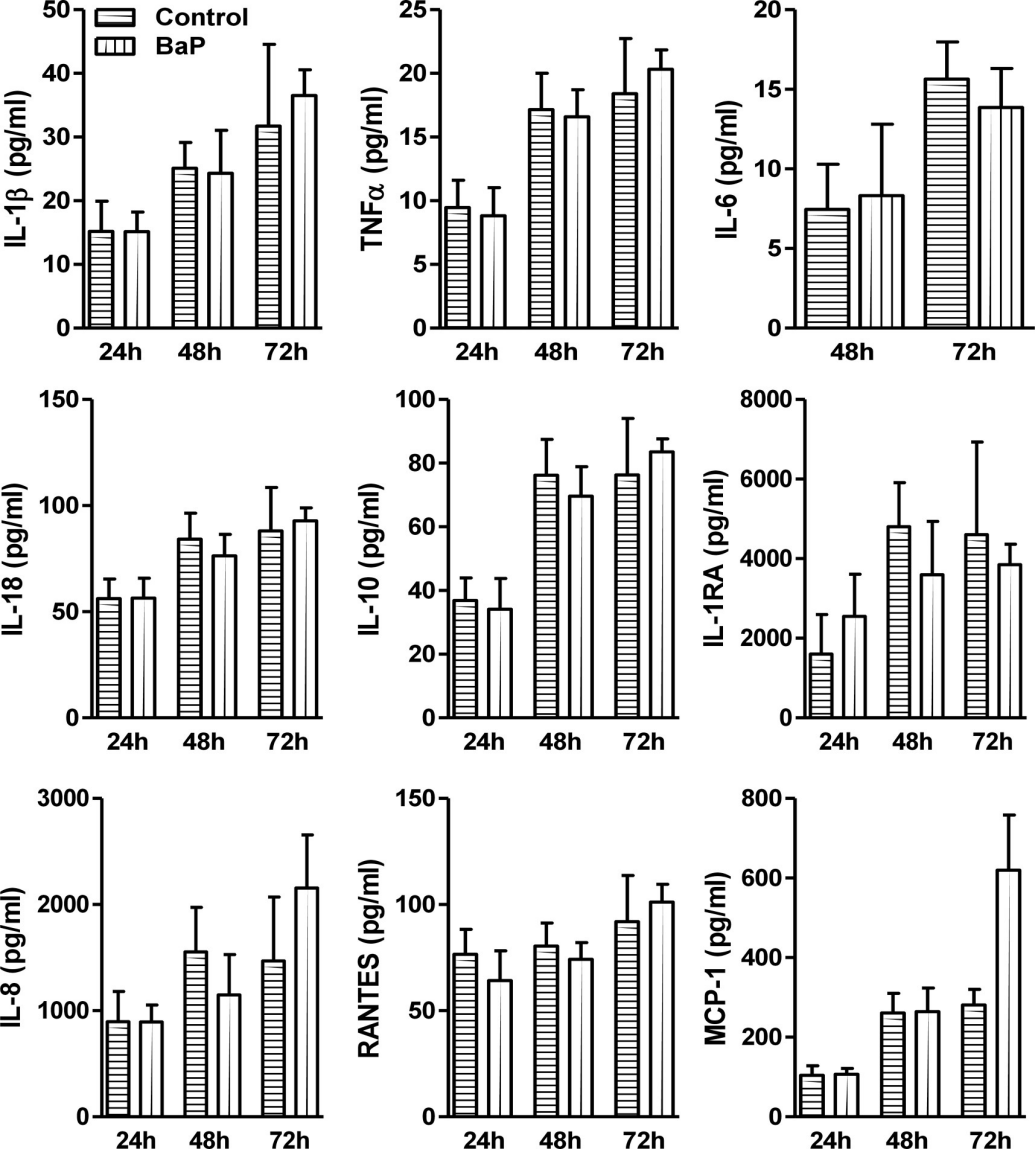
Levels of cytokines and chemokines in BaP-treated U1 cells. Multiplex ELISA analyses of pro-inflammatory cytokine (IL-1β, TNFα, IL-6, IL-18), anti-inflammatory cytokine (IL-10, IL-1RA), and chemokines (IL-8, RANTES, MCP-1) were performed in U1 cells (at 24, 48, and 72 h) treated with 1 μM concentration of BaP (daily treatment). Statistical analyses were carried out by using Student’s t-test. Results are expressed as means ± S.E.M of n = 3–4 experiments.

To determine whether BaP alters the cytokines and chemokines, we measured the extracellular release of proinflammatory cytokines (IL-1β, TNFα, IL-6, IL-18), anti-inflammatory cytokines (IL-10, IL-1RA), and chemokines (IL-8, RANTES, MCP-1) in BaP (1 μM) treated U1 cells supernatant. Our results demonstrated not much change in the levels of cytokines and chemokines in U1 cells supernatants treated with BaP at 24, 48, and 72 h (Fig 3). However, we noticed an interesting increase in the levels of MCP-1 as well as a modest increase in TNFα, IL-8 and IL-1β levels observed at 72 h of BaP treatment but could not reach to statistically significant level.

### BaP induces IL-1β expression in SVGA cells

**Fig 4:**
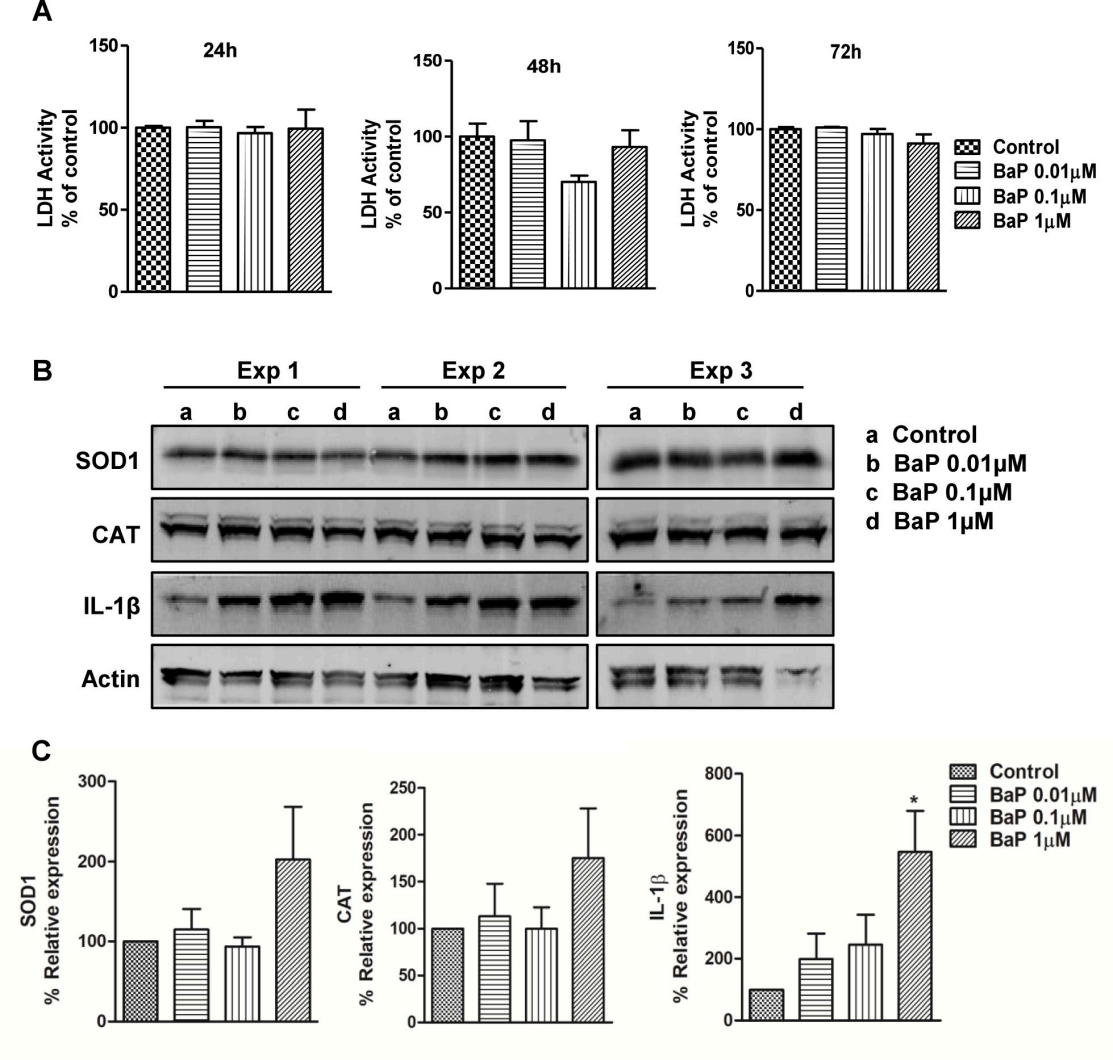
LDH activity and protein expression of AOEs and cytokine IL-1β (A, B). (A) LDH activity was performed in SVGA cells treated with different concentrations of BaP (daily treatment up to 72 h). (B) Representative Western blots of AOEs (SOD1 and CAT) and IL-1β in SVGA cells (Western blot at 72 h) treated with different concentrations of BaP (daily treatment up to day 3). Untreated controls were considered as 100% in the densitometry analysis. (C) Densitometry analysis. Statistical analyses were carried out by using one-way ANOVA. Results are expressed as means ± S.E.M of n = 4 experiments. *P ≤0.05, in comparison with untreated control.

Having observed an upward trend in the expression of SOD1, CAT, and IL-1β upon treatment with BaP in the U1 macrophages, we set out to investigate changes of SOD1 and CAT involved in the clearance of oxidative stress and IL-1β involved in pro-inflammatory immune response induced by BaP in SVGA cells. We treated the SVGA cells with different concentrations of BaP (0.01, 0.1, 1 μM) every 24 h up to 72 h to determine the effect of BaP on AOEs (SOD1 and CAT), and pro-inflammatory cytokine IL-1β. Before analyzing AOEs and IL-1β in SVGA cells upon BaP treatment, we performed an LDH assay to analyze whether BaP causes cell cytotoxicity. We observed that treatment of SVGA cells with BaP for 24, 48, and 72 h did not show a significant increase in LDH activity when compared to untreated cells (Fig 4A), indicating no detectable cytotoxicity with the BaP in SVGA cells. Though not statistically significant, we observed an increase in the expression of SOD1 and CAT at a 1 μM concentration of BaP (Fig 4B and 4C, S2 Fig). Notably, cellular expression of IL-1β was dose-dependently elevated by BaP treatment in SVGA cells. In addition, at 1 μM concentration, BaP significantly increased IL-1β protein (*P* ≤0.05; ANOVA; Fig 4C) expression in SVGA cells when compared to untreated cells.

### Time-dependent effect of BaP on IL-1β, AOEs, and CYP enzymes in SVGA cells

**Fig 5:**
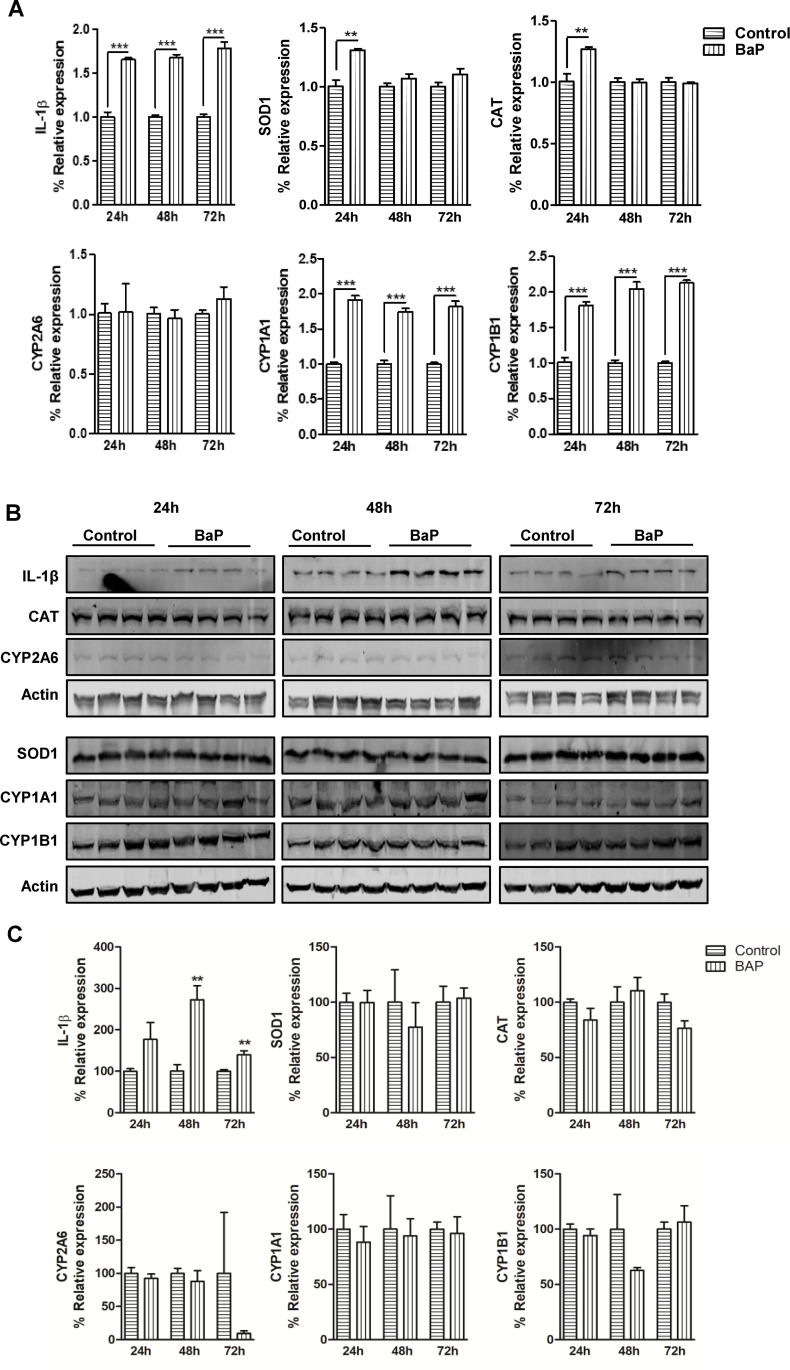
mRNA and protein expression of CYP enzymes, AOEs and cytokine IL-1β (A-C). (A) mRNA expression was analyzed in SVG cells (at 24, 48, and 72 h) treated with 1 μM concentration of BaP (daily treatment). (B) Representative western blots of IL-1β, AOEs (SOD1 and CAT), and CYP enzymes (CYP2A6, CYP1B1, CYP1A1) in SVG cells (Western blot at 24, 48, and 72 h) treated with 1 μM concentration of BaP (daily treatment). Untreated controls were considered as 100% in the densitometry analysis. (C) Densitometry analysis. Statistical analyses were carried out by using Student’s t-test. Results are expressed as means ± S.E.M of n = 4 experiments. **P ≤0.01, ***P ≤0.001 in comparison with untreated control.

Having demonstrated that BaP dose-dependently induces intracellular levels of IL-1β, a cytokine associated with a pro-inflammatory immune response, we then set out to evaluate the changes in the IL-1β expression at mRNA and protein levels in SVGA cells at different time points. Moreover, we analyzed the mRNA and protein expression levels of AOEs (SOD1 and CAT) and CYP enzymes (CYP2A6, CYP1A1, and CYP1B1), which are known to increase oxidative stress upon tobacco exposure, in SVGA cells at different time points (Fig 5A and 5B) after BaP (1 μM) exposure (Fig 5A–5C). IL-1β mRNA expression was significantly upregulated in SVGA cells at 24 h (P ≤0.001; Student’s t-test; Fig 5A), 48 h (P ≤0.001; Student’s t-test; Fig 5A) and 72 h (P ≤0.001; Student’s t-test; Fig 5A) post-BaP treatment when compared to untreated cells. Similarly, CYP1A1 and CYP1B1 mRNA expression was also increased after the BaP treatment at 24 h (P ≤0.001; Student’s t-test; Fig 5A), 48 h (P ≤0.001; Student’s t-test; Fig 5A) and 72 h (P ≤0.001; Student’s t-test; Fig 5A). Whereas SOD1 and CAT mRNA expression was found to have notably increased at 24 h (P ≤0.01; Student’s t-test; Fig 5A) only. However, CYP2A6 mRNA expression was not altered at any time point after the BaP treatment. Next, the protein expression of IL-1β, AOEs, and CYP enzymes was measured in SVGA cells treated with BaP (1 μM) at 24, 48, and 72 h (Fig 5B and 5C). IL-1β protein expression was significantly increased in SVGA cells at 48 h (*P* ≤0.01; Student’s t-test; Fig 5B and 5C, S3 Fig) and 72 h (*P* ≤0.01; Student’s t-test; Fig 5B and 5C, S3 Fig) post-BaP treatment when compared to untreated cells. Together, these data indicate that BaP exposure results in a significant increase in IL-1β expression in SVGA cells during the acute cellular phase. Moreover, we performed a western blot of AOEs (SOD1 and CAT) and CYP enzymes (CYP2A6, CYP1A1, and CYP1B1) in SVGA cells after BaP treatment for different time points (Fig 5B and 5C, S3 Fig). However, none of these were altered after the BaP treatment observed up to 72 h.

### Effect of BaP on Reactive Oxygen Species (ROS) in SVGA cells

**Fig 6:**
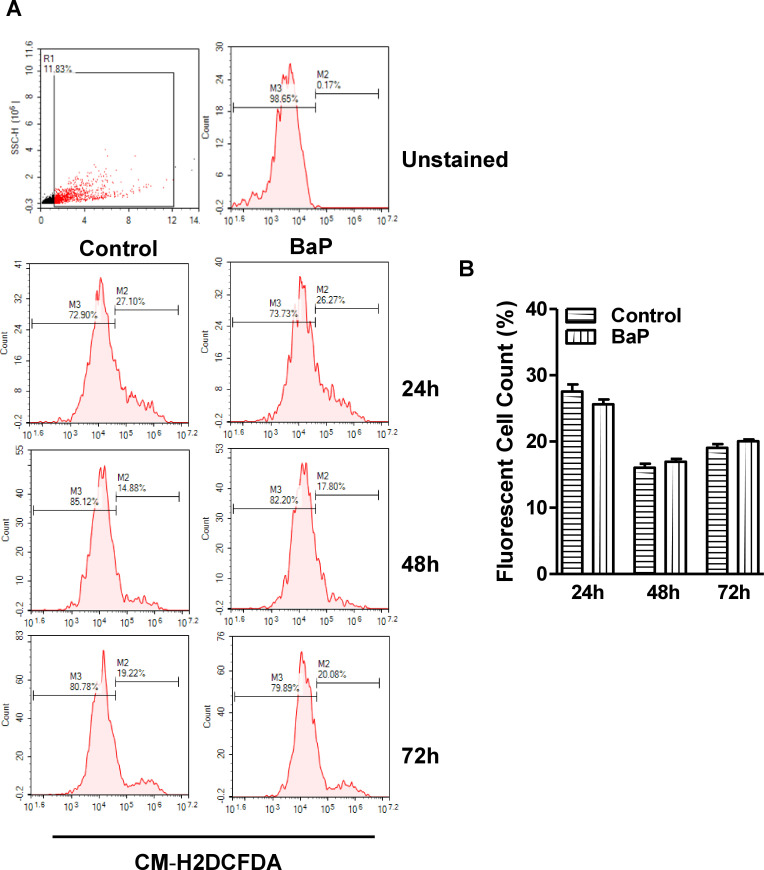
Effect of BaP on ROS in SVG cells (A, B). (A) The SVG cells were treated with 1 μM BaP for 24, 48, and 72 h. ROS level using flow cytometry. (B) Data is quantified using florescent cell count that were measured in %. Statistical analyses were carried out by using Student’s t-test (two groups each time point). Results are expressed as means ± S.E.M of n = 3 experiments.

Next, to examine the effect of BaP on ROS, SVGA cells were treated with 1 μM BaP for 24, 48, and 72 h. We analyzed the level of ROS by flow cytometry analysis. In the flow cytometry analysis, we did not see any significant changes in the ROS with BaP treatment up to 72 h (Fig 6A and 6B).

### BaP induces differential effects on cytokines/chemokines in SVGA cells

**Fig 7:**
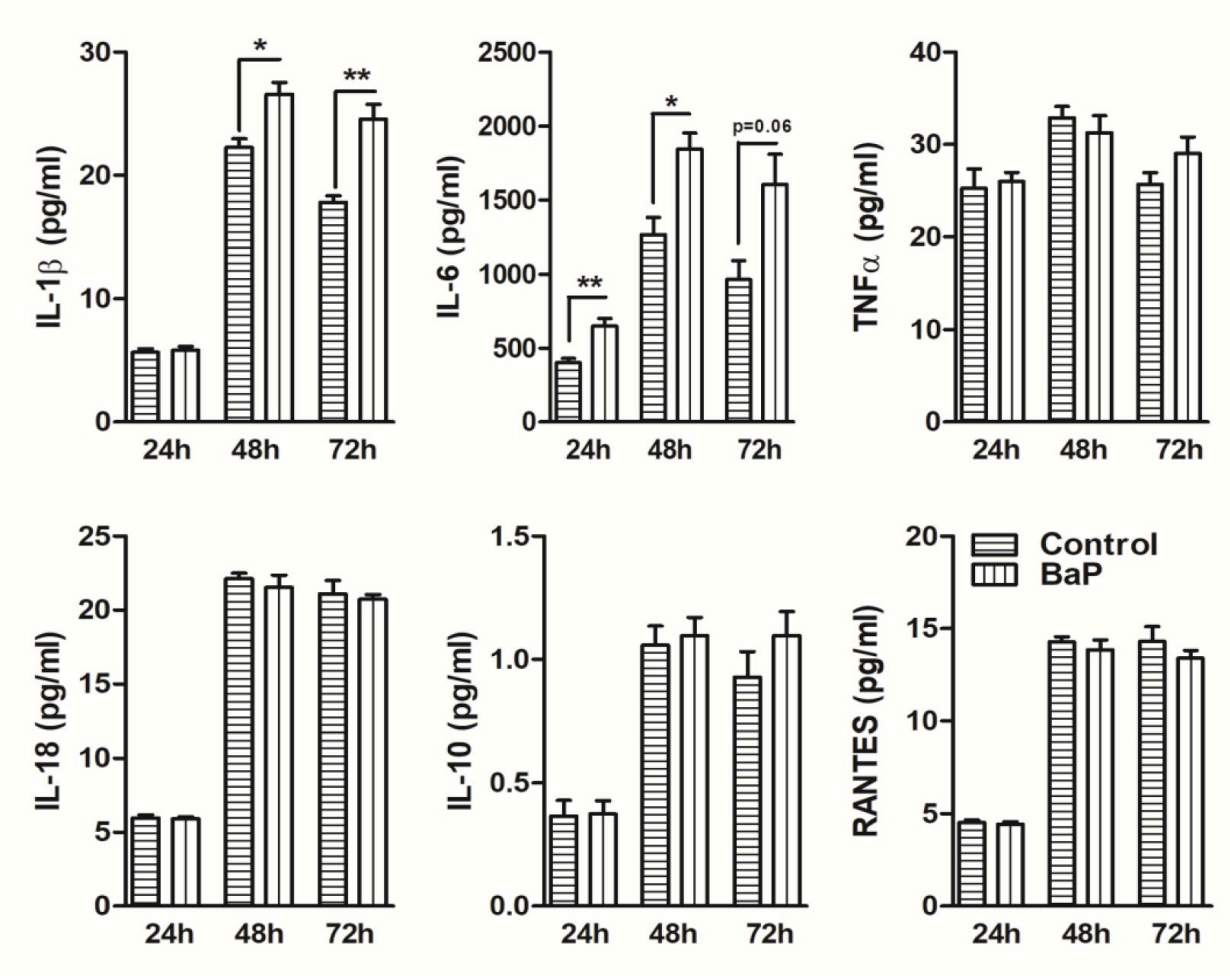
Levels of cytokines and chemokines in BaP-treated SVGA cells. Multiplex ELISA analyses of pro-inflammatory cytokine (IL-1β, IL-6, TNFα, IL-18), anti-inflammatory cytokine (IL-10), and chemokines (RANTES) were performed in SVG cells (at 24, 48, and 72 h) treated with 1 μM concentration of BaP (daily treatment). Statistical analyses were carried out by using Student’s t-test (two groups each time point). Results are expressed as means ± S.E.M of n = 3–4 experiments. *P ≤0.05 and **P ≤0.01 in comparison with untreated control.

Since IL-1β expression was enhanced in SVGA cells upon BaP treatment compared to control, we next investigated whether the release of other cytokines and chemokines also altered after BaP treatment. Several cytokines and chemokines have been reported to be altered in smokers with and without systemic and brain diseases [28, 29]. Therefore, we assessed nine cytokines/chemokines (IL-1β, IL-6, TNFα, IL-18, IL-8, IL-1RA, IL-10, RANTES, MCP-1) expression profiles in BaP (1 μM) treated SVGA cells supernatant at 24, 48, and 72 h. We observed that BaP significantly elevated IL-1β levels at 48 h (*P* ≤0.05; Student’s t-test; Fig 7) and 72 h (*P* ≤0.01; Student’s t-test; Fig 7) when compared to respective control cells. Notably, IL-6 cytokine, which is known to play a dual role during infections, was robustly increased in SVGA cells upon BaP treatment at 24 h (*P* ≤0.01; Student’s t-test; Fig 7) and 48 h (*P* ≤0.05; Student’s t-test; Fig 7). However, at 72 h, the IL-6 level was not found statistically significant, although an upward trend was observed. Furthermore, exposure of BaP to SVGA cells did not significantly alter the levels of TNFα, IL-18, IL-10, and RANTES at either 24, 48, or 72 h when compared to respective controls. IL-8, IL-1RA, and MCP-1 levels were not found in the detectable range.

## Discussion

Our prior findings have shown that cigarette smoke condensate (CSC) exposure enhanced HIV viral load and oxidative stressin HIV-infected monocytes/macrophages [30, 31]. Further, we noticed an increase in HIV viral load, oxidative stress, and nicotine metabolism with possible involvement of CYP enzymes in *ex vivo* samples obtained from HIV-infected smokers [30]. Taken together, these studies suggest an association of CYP-mediated nicotine metabolism and subsequent oxidative stress with HIV replication in tobacco smokers. A comparison of oxidative stress levels after nicotine and CSC exposure revealed that the induction of ROS by CSC is much higher than ROS induction by nicotine [11, 15, 31], suggesting the involvement of other PAH components of CSC such as BaP in the induction of oxidative stress. We recently studied the chronic effect of BaP, an important PAH component of CSC, in HIV infected monocytes/macrophages cells and observed that BaP increases CYP1A1 expression, ROS levels, and cytotoxicity [16, 23]. The excessive ROS production caused by BaP likely disturbed the redox homeostasis, causing oxidative stress, which resulted in cytotoxicity in U937 cells [16]. Furthermore, we demonstrated that oxidative stress generated by the CYP1A1-mediated metabolism of BaP, triggers the redox-sensitive transcription factor, NF-κB that leads to HIV replication [16]. In the present study, we have demonstrated the effect of BaP on the AOEs and cytokines/chemokines as inflammatory mediators in U1 and macrophages neighboring SVGA cells. Overall, our findings suggest that BaP can enhance ROS in U1 macrophages and inflammatory modulators, in particular, IL-1β in SVGA cells, which may potentially contribute to the tobacco/smoking-mediated neuroinflammation.

Upon exposure to smoking/tobacco products, cells activate their antioxidant mechanism to counteract oxidative stress [32, 33]. Oxidative stress induced due to an imbalance of free radicals/ROS and antioxidants has been implicated in the pathogenesis and progression of inflammatory diseases and HIV [34, 35]. Oxidative stress is alleviated by activation of an antioxidant defense mechanism that involves activation of antioxidant-related pathways, production of non-enzymatic antioxidants, and induction of antioxidant enzymes [36]. Activation of the transcription factor Nrf2 via the Keap1-Nrf2-ARE signaling pathway regulates the basal and inducible expression of AOEs (SOD, CAT, GPx) and counteract ROS levels to maintain physiological homeostasis [36]. Although under normal physiological conditions, these AOEs exert protective function against the adverse effects of xenobiotics, any perturbations in AOE function or ROS generation could lead to oxidative stress [37]. In many pathophysiological conditions, including cigarette smoking, excessive production of reactive species may accelerate the inactivation of nitric oxide and cause endothelial dysfunction that leads to inflammation, loss of vasodilation, platelet aggregation, and impaired modulation of vascular growth, and dysregulation of vascular remodeling [38]. While at the physiological level, ROS participates in various cellular processes including adaptation to hypoxia and regulation of autophagy, immunity, differentiation, and longevity [39]. Antioxidant functions are mainly dominated by three key enzymes SOD1, CAT, and glutathione peroxidase [40, 41]. In our previous studies [16, 23], we used low (100 nM for 7 days) and high (1 μM for 3 days) concentrations of BaP to study its effects on HIV replication and oxidative stress pathway in U1 and U937 cells. We showed that while chronic exposure to BaP at 100nM induced HIV replication in U1 cells, it failed to induce the oxidative stress mediators SOD1 and CAT at mRNA and protein levels.

In the present study, we investigated the acute inflammatory effects of up to 1 μM BaP in U1 macrophages and SVGA cells. Several prior in vitro and in vivo studies have used BaP concentration, which is comparable to or higher than 1 μM [23, 42–47]. Although 1 μM is higher than the physiological BaP brain concentration in humans, relatively high concentrations are routinely used to determine acute effects of BaP. A relatively high BaP concentration in acute treatment may correlate to near physiological BaP concentration in a particular region of the brain in heavy and chronic cigarette smokers. Therefore, the concentration of BaP 0.01–1 μM up to 3 days (acute treatment) was chosen in our study to investigate the effects of BaP on oxidative stress and inflammatory mediators in U1 and SVGA cells. Moreover, BaP 1 μM concentration is toxic to the cells when treated for 7 days. Consistent with the previous findings, even at 1 μM BaP for 3 days, we observed no significant alteration in the mRNA expression of SOD1 and CAT in U1 macrophages. However, an upward trend was observed in the protein expression of SOD1 and CAT, suggesting mRNA decay or post-transcriptional modification. Possibly due to this increase in protein levels, we observed BaP-mediated production of ROS at early time points in U1 macrophages. In SVGA cells, we observed an increase in the mRNA expression of SOD1 and CAT at an early time point after BaP exposure, no significant changes were observed in the protein expression of SOD1 and CAT, which may be the reason associated with no effect on the production of ROS in SVGA. More, importantly, unlike in U1 cells, a significant increase in the IL-1β at both mRNA and protein levels in the SVGA astrocyte was observed. Our results suggest that BaP exposure in SVGA cells induces notable oxidative stress, and produces a strong pro-inflammatory activation state, similar to the immune response after a xenobiotic encounter in the brain. The results also suggest that astrocytic cells are relatively more susceptible to tobacco constituent, BaP, compared to macrophages.

Proinflammatory cytokines are believed to drive the neuroinflammatory events in many neurodegenerative diseases including HIV-associated neurocognitive disorder [48–50]. Cytokines including IL-1 levels have been found to be elevated in sera and monocytes/macrophages in HIV patients, and in the cerebrospinal fluid (CSF) of HIV patients suffering from HAND [51, 52]. During HIV infection, IL-1β and other cytokines has been inducted by HIV accessory protein TAT, in T cells and monocytes/macrophages, major target cells for HIV in the blood [53, 54]. Exposure of BaP induces ROS and IL-8 production in normal human epidermal keratinocytes through the activation of the aryl hydrocarbon receptor signaling pathway [55], whereas the production of IL-1α, IL-6, TNF-α, and GM-CSF was not affected by the BaP. Another study also suggests that BaP exacerbates colonic damage via inducing TNF-α and IL-1β expression in the mucosa of mice challenged with BaP (62.5mg/kg-250mg/kg) when compared with control [56]. In our study, IL-1β produced from the BaP (1 μM) treated SVGA cells was found to be increased substantially than untreated cells. The mRNA expression was found significantly increased at 24, 48, and 72 h, whereas the magnitude of IL-1β protein expression at intracellular levels was found significantly increased at 48 h and 72 h post-treatment with BaP, suggesting that BaP treatment causes upregulation of the expression of IL-1β. These data clearly show that following BaP treatment, IL-1β is induced within SVGA cells which results in an increased inflammatory milieu inside the cells. Though, the expression of SOD1 and CAT were not found significantly altered at any time point of investigation.

The current study also investigated whether BaP-induced pro-inflammatory cytokine IL-1β in SVGA cells is consistent with the induction of CYP enzymes *in vitro*. In a prior study, we have shown that CSC exposure increases oxidative stress and induction of CYP enzymes in U937 and/or U1 macrophages [31]. CYP enzyme, in particular, CYP2A6 was shown to be involved in nicotine metabolism and oxidative stress in U937 cells [11]. These studies suggested the involvement of CYP enzymes in the production of oxidation stress and increased HIV viral load in HIV-infected smokers. A prior study also showed that BaP (5 μM) increases the expression of CYP enzymes (CYP1A1 and CYP1B1), production of ROS, and cytotoxicity in lung epithelial cells (BEAS-2B cells) after 24 h treatment [57]. It has been earlier shown that CYP enzymes regulate the production of ROS via several mechanisms that include regulation of gene transcription and modulation of interactions between protein components of the p450 monooxygenase resulting in its poor activity, coupling, and stability [58]. We earlier studied the effect of BaP in U937 and/or primary monocytic cells and observed that BaP (100nM) increases cytotoxicity, which is likely through the CYP1A1 and oxidative stress mediators [16]. Recently, we investigated whether BaP affects HIV viral load and induced the oxidative stress pathway via the CYP pathway *in vitro* [23] and observed that BaP enhances the HIV replication in macrophages by a CYP-mediated oxidative stress pathway followed by the NF-κB pathway. Furthermore, a significant increase was observed in the gene expression of CYP1A1 in addition to enhanced ROS production and cytotoxicity in U1 cells. These findings suggest that BaP-induced ROS occurs via CYP1A1-mediated metabolic activation of BaP [23]. A study performed in a human lung cell line also indicated a significant role of CYP1A1 in the formation of BaP to its hydrolyzed carcinogenic form BaP-diol-epoxides, while both CYP1A1 and CYP1B1 were found to be involved in the metabolism of BaP [59]. Therefore, in the present study, we chose to investigate the BaP effect on the induction of CYP enzymes. The expression of CYP2A6, CYP1A1, and CYP1B1, which are the major tobacco metabolizing CYPs, were examined in SVGA cells after BaP (1 μM) exposure daily for 72 h. The result did not show significant changes in the mRNA and protein expression of CYP2A6. At the mRNA level, we did see significant changes in the expression of CYP enzymes (CYP1A1 and CYP1B1) in SVGA cells upon BaP treatment, which are potentially associated with the BaP effects on IL-1β. However, we did not observe the significant expression of CYP1A1 and CYP1B1 at protein levels, perhaps due to post translational modifications or instability of the protein after extraction. The discrepancy in these results with our earlier investigation could be due to the use of different, monocytic (U937 and U1) and astrocytic (SVGA) cell lines. Chi et al, in their study, showed similar CYP1A1/CYP1B1 induction or preferential CYP1A1 induction in tissue-specific tobacco-related squamous cell carcinoma cell lines [60]. In contrast to their investigation of CYP enzymes in cell lines, their investigation of gingival tissue revealed preferential induction of CYP1B1 among smokers [60]. Thus, monocyte and SVGA astrocytes may show differential inflammatory responses and expression of CYP enzymes in response to BaP exposure. In the present study though not at the protein level, we did see an increase in mRNA levels of CYP1A1 and CYP1B1 possibly suggesting involvement of CYPs in the BaP-induced IL-1β production in SVGA cells. There are no changes observed on ROS upon BaP treatment in SVGA cells possibly associated with no change in the levels of AOEs at protein levels. Other than the expected release of IL-1β upon BaP treatment in SVGA cells, the release of other cytokines mainly IL-6 was also found to be increased. A prior study showed that CYP1A1 overexpression augmented TNF-α and IL-6 production in RAW264.7 cells by enhancing JNK/AP-1 signaling [61]. Other studies also suggested the involvement of JNK/AP-1/ATF2 and NF-kB/IKK-β signaling in the production of IL-6 in microglia and monocytes/macrophages, respectively [62–64]. However, further study is needed to examine the mechanism of IL-1β and IL-6 production in the context of BaP and SVGA cells. Future studies could be conducted on other glial cells to determine the comprehensive role of BaP on neuroinflammation. Overall, in our study we showed the effect of BaP on the astrocytes and macrophages that BaP predominantly induce IL-1β production and presents clinical application of cytokine therapy in combination with cART that may play an important role in offering new strategies for modulation and treatment of smoking-mediated HIV pathogenesis and HIV-associated neuropathogenesis.

## Supporting information

S1 Fig Original western blot images for Fig 1B. (PDF)

S2 Fig Original western blot images for Fig 4B. (PDF)

S3 Fig Original western blot images for Fig 5B. (PDF)
