# Supplementary figures and images for: Effect of benzo(a)pyrene on oxidative stress and inflammatory mediators in astrocytes and HIV-infected macrophages
Source: PLoS One. 2022 Oct 14;17(10):e0275874. doi: 10.1371/journal.pone.0275874 (PMC9565757; doi:10.1371/journal.pone.0275874)

Fig 1B. Original blots

a Control  
b BaP 0.01 $\mu$ M  
c BaP 0.1 $\mu$ M  
d BaP 1 $\mu$ M

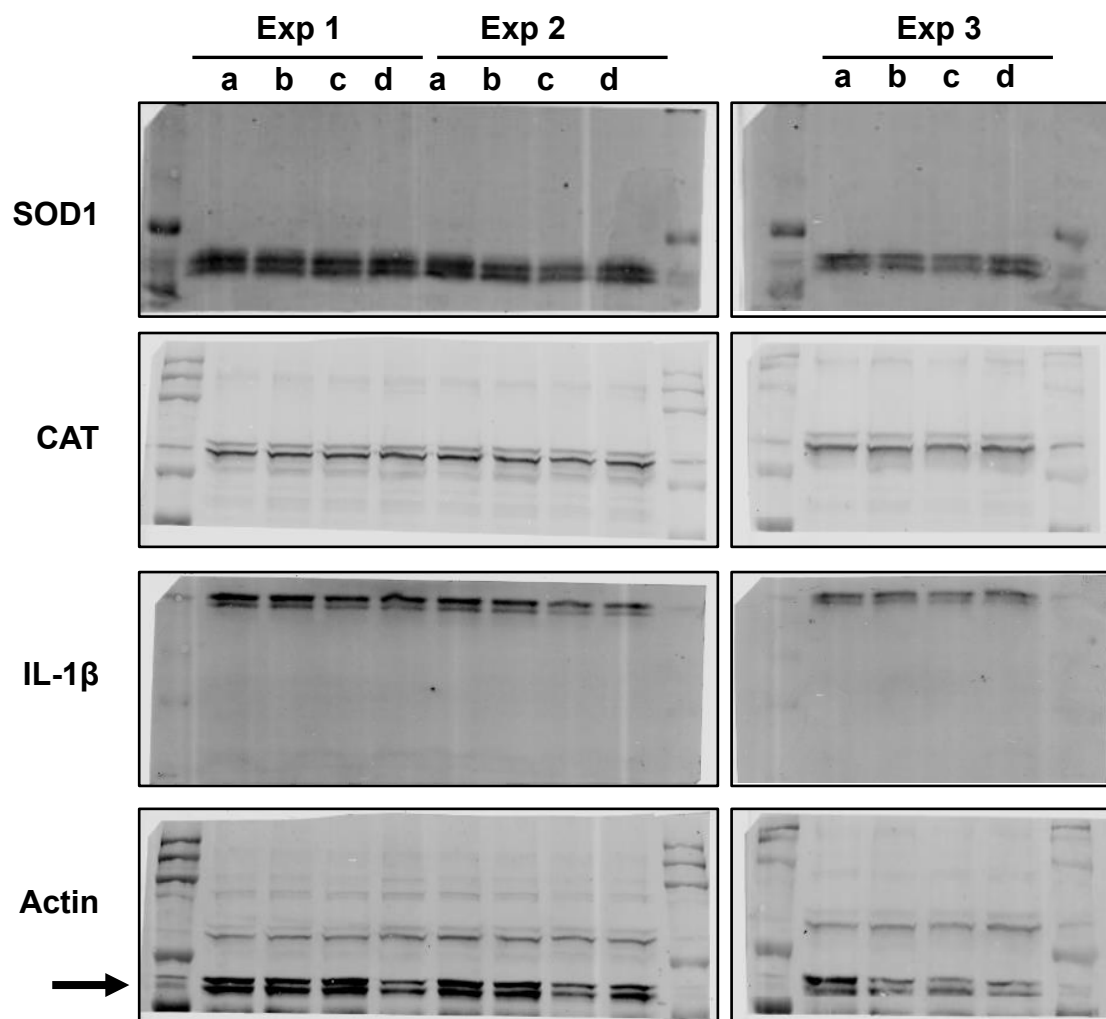

Suppl. Fig. 1

Supplement: S1 Fig — (PDF) [file pone.0275874.s001.pdf]

Fig 4B. Original blots

a Control  
b BaP 0.01μM  
c BaP 0.1μM  
d BaP 1μM

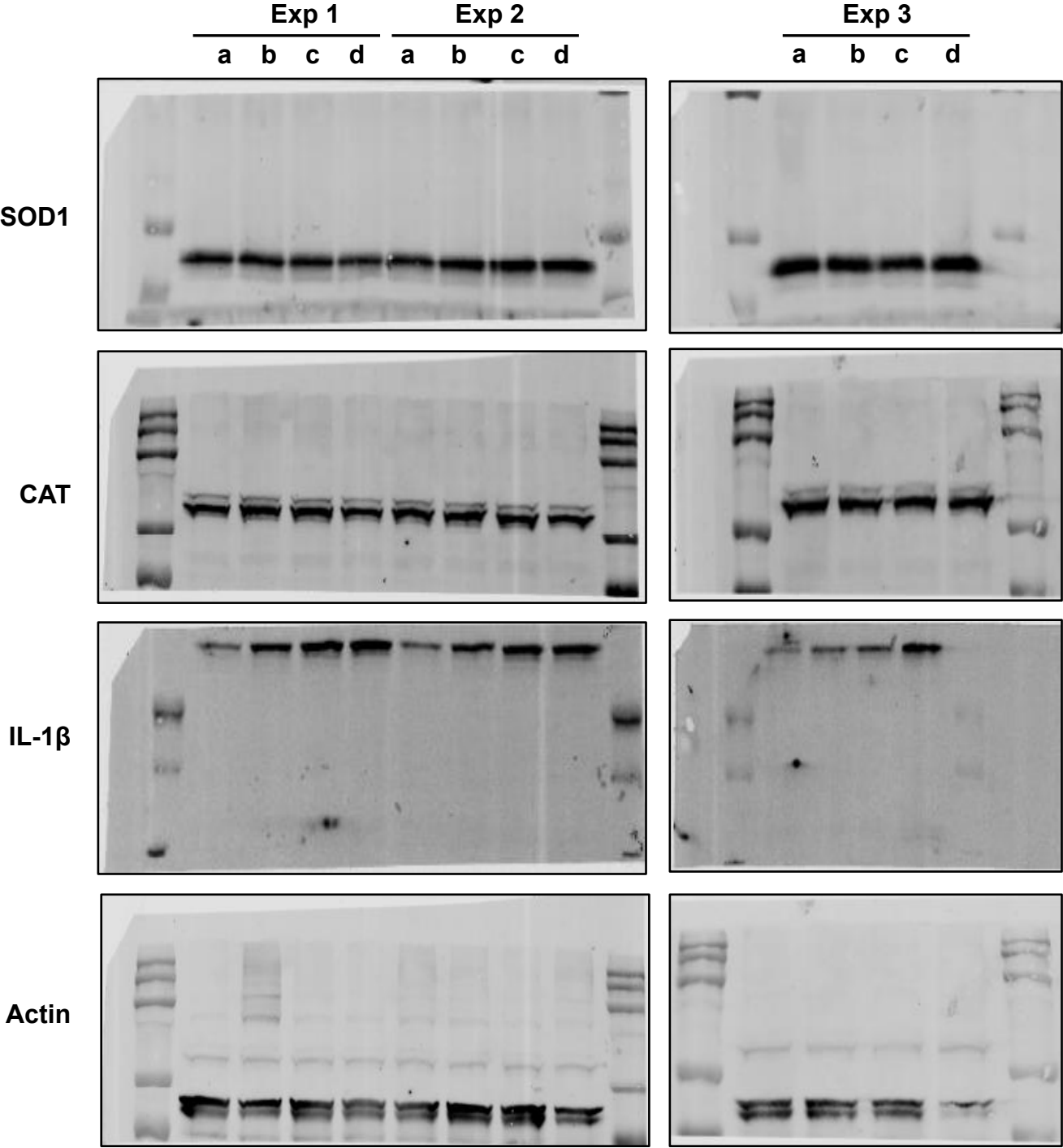

Supplement: S2 Fig — (PDF) [file pone.0275874.s002.pdf]

Fig 5B. Original blots

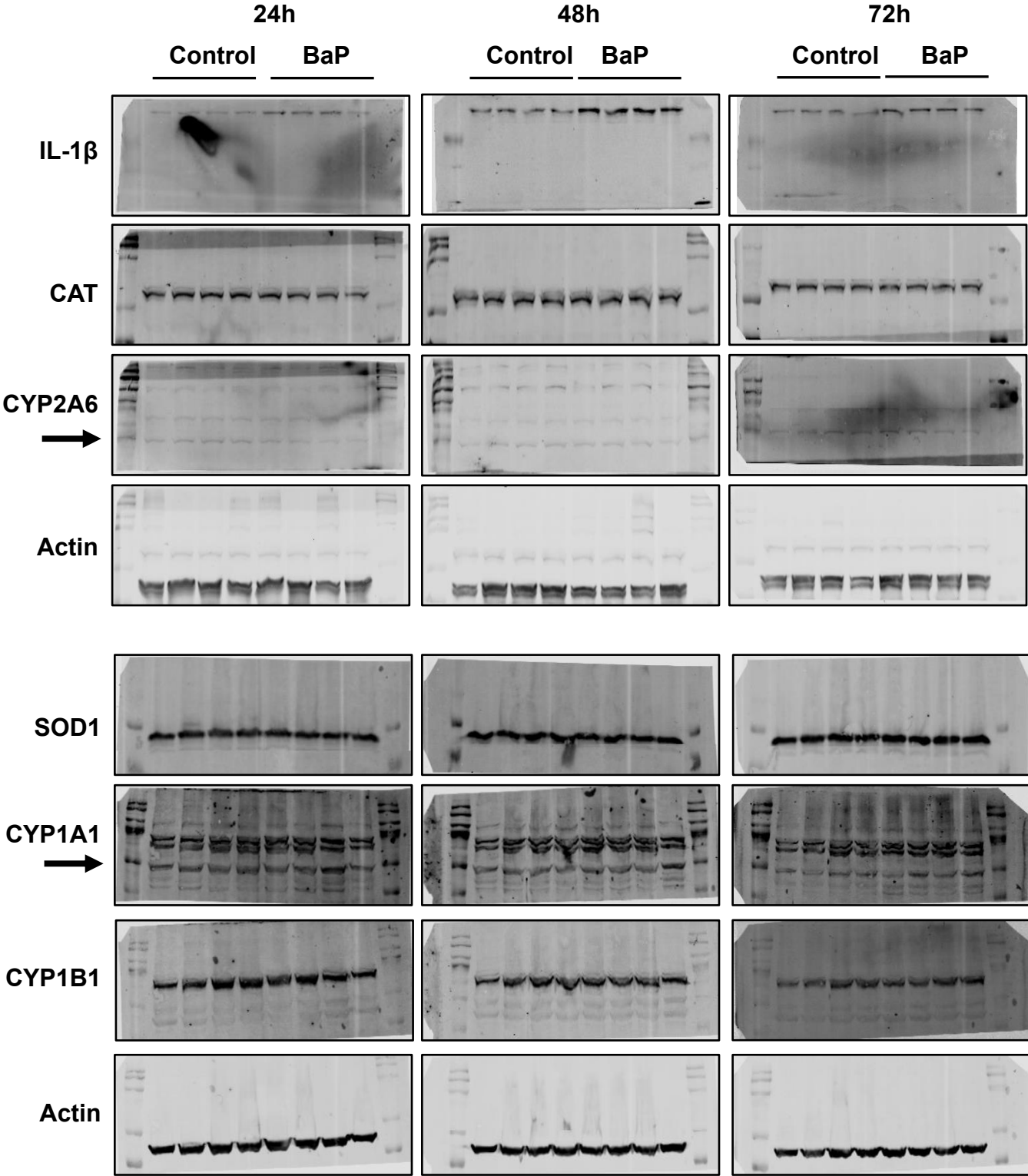

Supplement: S3 Fig — (PDF) [file pone.0275874.s003.pdf]
